# Vanadium Carbide (V_4_C_3_) MXene as an Efficient Anode for Li-Ion and Na-Ion Batteries

**DOI:** 10.3390/nano12162825

**Published:** 2022-08-17

**Authors:** Qiong Peng, Javed Rehman, Kamel Eid, Ayman S. Alofi, Amel Laref, Munirah D. Albaqami, Reham Ghazi Alotabi, Mohamed F. Shibl

**Affiliations:** 1 Institution of Condensed Physics & College of Physics and Electronics Engineering, Hengyang Normal University, Hengyang 421002, China; 2 Department of Physics, Balochistan University of Information Technology, Engineering and Management Sciences (BUITEMS), Quetta 87300, Baluchistan, Pakistan; 3 Gas Processing Center (G.P.C.), College of Engineering, Qatar University, Doha 2713, Qatar; 4 Physics Department, College of Science, Taibah University, Medina 42353, Saudi Arabia; 5 Department of Physics and Astronomy, College of Science, King Saud University, Riyadh 11451, Saudi Arabia; 6 Chemistry Department, College of Science, King Saud University, Riyadh 11451, Saudi Arabia; 7 Center for Sustainable Development, College of Arts and Sciences, Qatar University, Doha 2713, Qatar

**Keywords:** V_4_C_3_, M*X*ene, Li-ion battery, Na-ion battery, electrochemical energy storage, DFT

## Abstract

Li-ion batteries (LIBs) and Na-ion batteries (SIBs) are deemed green and efficient electrochemical energy storage and generation devices; meanwhile, acquiring a competent anode remains a serious challenge. Herein, the density-functional theory (DFT) was employed to investigate the performance of V_4_C_3_ MXene as an anode for LIBs and SIBs. The results predict the outstanding electrical conductivity when Li/Na is loaded on V_4_C_3_. Both Li_2*x*_V_4_C_3_ and Na_2x_V_4_C_3_ (*x* = 0.125, 0.5, 1, 1.5, and 2) showed expected low-average open-circuit voltages of 0.38 V and 0.14 V, respectively, along with a good Li/Na storage capacity of (223 mAhg^−1^) and a good cycling performance. Furthermore, there was a low diffusion barrier of 0.048 eV for Li_0.0625_V_4_C_3_ and 0.023 eV for Na_0.0625_V_4_C_3_, implying the prompt intercalation/extraction of Li/Na. Based on the findings of the current study, V_4_C_3_-based materials may be utilized as an anode for Li/Na-ion batteries in future applications.

## 1. Introduction

The everlasting consumption of fossil fuels leads to their depletion and greenhouse gas emissions, which are the primary cause of global warming [1,2,3]. A variety of endeavors are currently being dedicated to addressing these issues, including gas conversion reactions [4,5] and utilizing sustainable energy sources (i.e., solar power [6,7], hydrogen power [8], fuel cells [9,10], and energy storage devices [11,12,13,14,15]). Li-ion batteries (LIBs) and Na-ion batteries (SIBs), with their high energy, power density, and long cycle life, are among the most beneficial electrochemical energy conversion and storage technologies available for smart grids, mobile electronics, and electric vehicles [16,17,18]. The performance of LIBs and SIBs is primarily shaped by the electrochemical properties of the anode materials [16,17]. Graphitic carbon is the universally utilized commercial anode material, but its low Li/Na theoretical capacity (372/25 mAh/g) and low rate capability limit its widespread, practical use [19]. Despite the significant progress in LIBs and SIBs, the earth availability of Li/Na, charge time, durability, temperature tolerance, self-discharge, and recyclability of the decayed batteries are creating a significant challenge [16,17,18,19,20,21,22]. Therefore, developing novel anodes with high specific capacities, greater rate capabilities, and cycling longevity is imperative.

MXenes are a novel class of 2D transition metal carbide/carbonitride electrodes that have several advantages for LIBs, SIBs, and other applications, including hydrophilicity, high active surface areas, rich electron densities, and low costs [23,24,25]. Numerous MXenes such as Ti_2_C, Ti_3_C_2_, V_2_C, Nb_2_C, and Mo_2_C were utilized as anodes for LIBs, and SIBs with the Ti_3_C_2_ MXene phase has been studied most extensively [23,24,25,26,27,28]. Distinct from other MXenes, V_4_C_3_ MXene offers many advantages, including greater interlayer spacing, better structural durability, and high specific capacity, which are essential for the fabrication of high-performance anodes for LIBs and SIBs [29,30,31]. Besides its excellent mechanical properties and thermal stability, V_4_C_3_ MXene possesses excellent metallic properties due to its narrow band gap at the Fermi level [32,33]. Meanwhile, the vanadium metal (V) in V_4_C_3_ MXene has a prosperous valence state from +2 to +5, which may enhance the electrochemical performance of LIBs and SIBs [29,34,35]. For instance, the V_4_C_3_ MXene/MoS_2_/C electrode significantly boosted LIB activity compared to MoS_2_/C and MoS_2_ electrodes, showing an outstanding reversible capability of 0.622 Ah/g at 1 A/g after 450 cycles and maintaining a superior rate capability of 0.5 Ah/g at 10 A/g [36]. That is due to the outstanding electrical conductivity, structural durability, and fast reaction kinetics promoted by V_4_C_3_. Likewise, V_4_C_3_T_x_ (T = O, OH, and F), which is formed by the ball milling (B.M.) of V_4_AlC_3_ followed by HF etching (V_4_C_3_T_x_-BM-HF), enhanced the LIB performance over V_4_C_3_T_x_-HF and yielded a specific capacity of 0.225 Ah/g after 300 cycles at 0.1 A/g and 0.125 Ah/g at 1/A g because of the superior interlayer spacing and specific surface area [37]. Despite the noted progress in V_4_C_3_ MXene, it is rarely reported on for applications in energy storage, and usually is exclusively with regard to LIBs; to the best of our knowledge, it has not been yet addressed theoretically for both LIBs and SIBs.

In pursuit of this aim, we employed the first principle, DFT simulation, to predict the performance of V_4_C_3_ MXene as an anode for LIBs and SIBs as a function of Li and Na loading. V_4_C_3_ MXene loaded with Li/Na was investigated for lithiation, sodiation, electrical conductivity, and surface energy. The surface energy is calculated by considering Li/Na loading on V_4_C_3_ with a diffusion barrier of 0.023 eV for Li and 0.048 eV for Na migration. 

## 2. Methodology

To conduct the current DFT investigations, we employed VASP software (Vienna, Austria) known as the Vienna *Ab Initio* Simulation Package [38], whereas correlation potential and the electronic exchange were examined by utilizing a generalized gradient (GGA) combined with a Perdew–Burke–Ernzerhof (PBE) functional (GGA-PBE). This is because the GGA-PBE is a nonempirical functional with judicious accuracy for qualitative and quantitative prediction of the molecules interacting and being stored with metal surfaces over a wide range of systems [39]. In the present calculations, we restricted the force value to 1/100 eV/Å, and the energy was 1 × 10^−6^ eV. Based on the GGA-PBE level, we simulated the electronic structure of V_4_C_3_ and Li/Na loaded V_4_C_3_. For plane-wave expansion, cut-off energy of 500 eV was selected. The Monkhorst–Pack technique was employed to sample the k-points in the Brillouin zone, with a dense k-point grid of 17 × 17 × 1 [40]. Additionally, the DFT-D2 model [41] was applied in our calculations to acquire reliable binding strength between Li/Na and V_4_C_3_. In the structure of V_4_C_3_, we generated a vacuum space of 20 Å to prevent coupling between V_4_C_3_ layers. 

Our simulations found that the materials under research are spin-polarized with Li/Na content loading. The voltage and energy profiles were computed with increasing Li/Na content, such that *x* = 0.125, 0.25. 0.5, 1.0, 1.5, and 2. The electronic structure calculations were carried out within the GGA-PBE to determine the electronic density of states (DOS). The AIMD simulations were used to investigate the change in the energy fluctuation of Li/Na-loaded V_4_C_3_ at 300 K within each time step of 1 fs for the total time duration of 5000 fs [42]. Several Li/Na concentrations were studied to procure the binding energies and voltage profile. The relationship of binding energy is shown in Equation (1) [43]:

(1) $$E_{b}=(E_{Li-V_{4}C_{3}\;}+nE_{Li}-E_{V_{4}C_{3}\;})/n,$$

where $E_{Li-V_{4}C_{3}\;}$ represents the Li-loaded V_4_C_3_ energy, $E_{V_{4}C_{3}\;}$ denotes the bare V_4_C_3_ energy, $E_{Li}$ is the metallic Li energy, and *n* is the number of Li content loaded on the V_4_C_3_ sheet. Similarly, we adopt the above formula for Na adsorption by substituting Li with Na to estimate *E*_b_. Next, we calculate the charge density difference based on the relation: $\Delta \rho \left(r\right)=\rho_{Li-V_{4}C_{3}}\left(r\right)–\rho_{V_{4}C_{3}}\left(r\right)–\rho_{Li}\left(r\right)$). Here, $\rho_{Li-V_{4}C_{3}}$ specifies the charge density of Li-loaded V_4_C_3_, $\rho_{V_{4}C_{3}}$ denotes the charge density of bare V_4_C_3_, and $\rho_{Li}$ is the charge density of Li (isolated). For Na-loaded V_4_C_3_, a similar formulation is employed by substituting only Li with Na. 

For each concentration of the Li*_x_*V_4_C_3_ compound, the open-circuit voltage (OCV) is evaluated by Equation (2) [44]:

(2) $$\;V\left(x1,\;x2\right)=\left[E_{Li_{x1}}-E_{Li_{x2}}+\left(x_{2}-x_{1}\right)\;E_{Li}\right]/\left(x_{2}-x_{1}\right)e$$

where $E_{Li_{x1}}$, $E_{Li_{x2}}$, and $E_{Li}\;$ are the energies of ${\mathrm{Li}}_{x1}$ V_4_C_3_, ${\mathrm{Li}}_{x2}$ V_4_C_3_, and bulk Li, respectively. A detailed discussion of the voltage profile is given in the supporting information. 

The theoretical capacity (C) can be determined through Equation (3):

(3) $$C\;=nF/M_{V_{4}C_{3}\;}$$

where n denotes the number of adsorbed Li/Na atoms, F defines the Faraday constant (26,801 mAh/mol), and $M_{V_{4}C_{3}\;}$ is the molar weight of V_4_C_3_.

The Bader charge technique was employed to calculate the amount of charge transferred from Li/Na to V_4_C_3_ (Table 1). Finally, the charging and discharging processes were investigated by using the simulation of surface barriers and minimum energy paths (MEPs) of Li/Na migration in the V_4_C_3_ monolayer with the climbing nudged elastic band (CI-NEB) method. This technique approximately justifies metal-ion batteries’ lithiation/delithiation and sodiation/desodiation mechanisms [45]. 

## 3. Results and Discussion

### 3.1. Structure of V_4_C_3_ Monolayer

**Figure 1:**
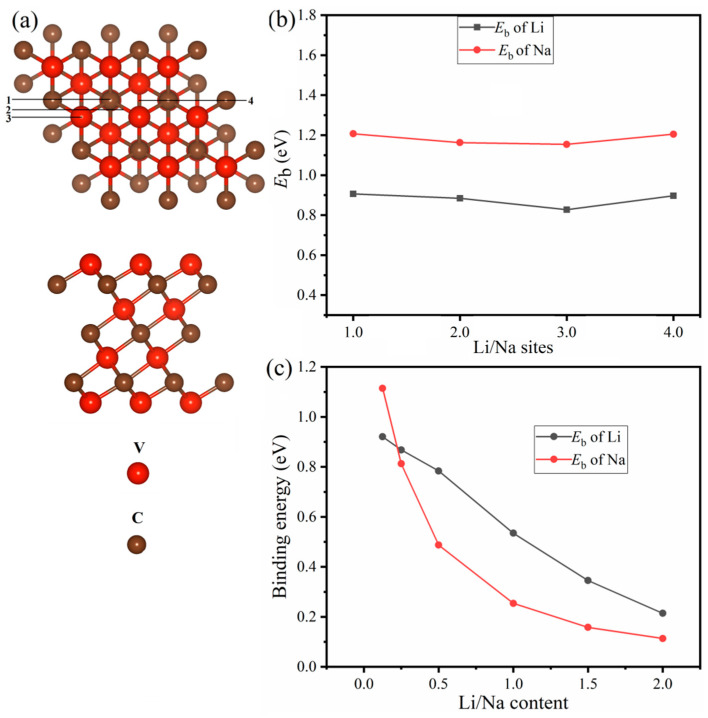
(**a**) Structural model of V_4_C_3_ *MX*ene with top and side views and (**b**) stable Li/Na sites with their *E*_b_ at *x* = 0.0625. (**c**) *E*_b_ with increasing Li/Na content. The numbers 1,2,3, and 4 represent the adsorbed four sites site-1, site-2, site-3, and site-4, respectively.

**Figure 2:**
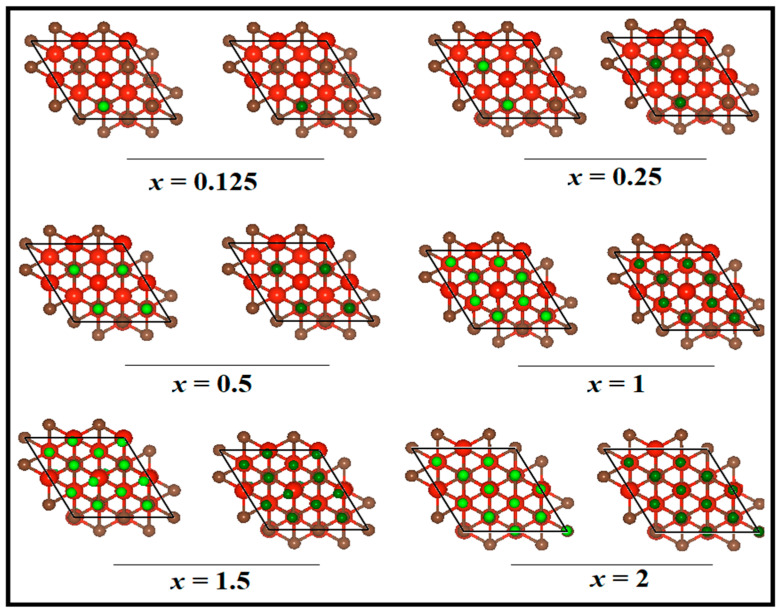
Front views of optimized structures of Li*_x_*V_4_C_3_ and Na*_x_*V_4_C_3_ at *x* = 0.125, 0.25, 0.5, 1, 1.5, and 2. The red color balls are V, brown ones are C, green ones are Li, and dark green ones are Na.

As a first step, we shall examine the structure of the V_4_C_3_ monolayer, which can be viewed in Figure 1a where the top and side views are shown. The structure portrays four layers of vanadium (V) and three layers of carbon (C) atoms. Each carbon layer is sandwiched between two V layers. In the relaxed structure, a unit-cell of V_4_C_3_ is composed of four V atoms and three C atoms with lattice parameters *a* = *b* = 2.90 Å and thickness *d* = 6.96 Å. These structural parameters are in line with the preceding results [46]. Currently, experimental data are available for the structure of V_4_C_3_ MXene; thus, it is interesting to investigate its anodic properties for LIBs and SIBs using DFT calculations. To determine the binding energies, the Li and Na are first adsorbed on V_4_C_3_ MXene. We selected four stable sites on the surface of V_4_C_3_ for Li/Na adsorption. The calculated *E*_b_ of the adsorbed four sites, site-1, site-2, site-3, and site-4 are 0.90 eV, 0.884 eV, 0.828 eV, and 0.897 eV, respectively, for Li (*x* = 0.0625). Similarly, for Na (*x* = 0.0625) adsorption, the binding energies are 1.21 (site-1) eV, 1.16 eV (site-2), 1.15 eV (site-3), and 1.20 eV (site-4) as depicted in Figure 1b. Comparatively, the adsorbed site-1 possesses greater binding energy for both Li/Na adsorptions. Thus, we picked site-1 for further adsorption of Li/Na loading. To avoid the repulsive interactions between Li^+^−Li^+^ and Na^+^−Na^+^, we consider that both surfaces (top/bottom) of V_4_C_3_ *MX*ene acquire reliable binding strength and maximum Li/Na ion storage. Figure 1c depicts the decreasing binding energy curves with increasing Li/Na concentrations at *x* = 2. A decreasing trend in *E*_b_ curves is noticeable due to the Li^+^−Li^+^ and Na^+^−Na^+^ repulsive forces. A similar pattern was also discerned in other 2D materials upon Li/Na loading [47,48]. The various optimized Li/Na-loaded content structures with front and side views are shown in Figure 2 and Figure S1, respectively. Subsequently, we found the amount of charge transferred from Li/Na to V_4_C_3_ by employing the Bader charge analysis. The amount of charge transfer from Li to V_4_C_3_ and Na to V_4_C_3_ is given in Table 1 [47,48,49]. A large amount of charge transfer from Li/Na to V_4_C_3_ confirms the binding energy curve (Figure 1c). The decrease in binding energy means there is a repulsion of charge due to Coulomb forces. It could be deduced from these results that there is a charge transfer from Li/Na to the V_4_C_3_ surface [47,48,49]. This reveals that an electrochemical reaction may occur between Li/Na and V_4_C_3_.

### 3.2. Safety and Stability of Li/Na-Loaded V_4_C_3_

**Figure 3:**
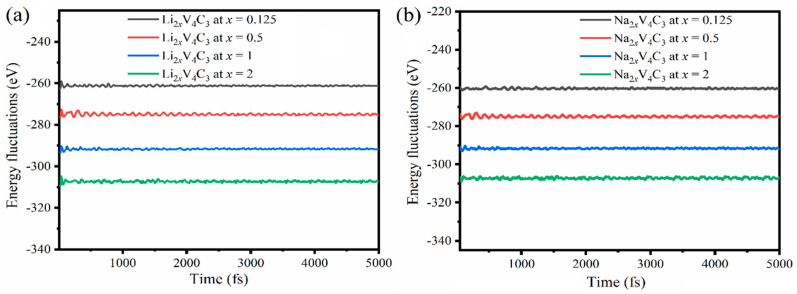
Energy fluctuations vs. time duration for (**a**) Li_2*x*_V_4_C_3_ and (**b**) Na_2*x*_V_4_C_3_ at *x* = 0.125, 0.5, 1, and 2.

Volume alteration of the V_4_C_3_ monolayer was studied in the in-plane expansion of the V_4_C_3_ single-layer (Figure S2) upon Li/Na adsorption. The results reveal that the lattice parameters increased with Li/Na adsorption increments in both Li_2x_V_4_C_3_ and Na*_2x_*V_4_C_3_, whereas the highest expected lattice expansions were about ~4.31% and 6.20%, respectively. Noticeably, V_4_C_3_ revealed a lower volume alteration during adsorption/desorption of Li/Na than graphite [50,51]. The energy fluctuation was computed and compared to time duration at 300 K (25 °C) using AIMD simulations to estimate the change in the structure of Li_2*x*_V_4_C_3_ and Na_2*x*_V_4_C_3_ (*x* = 0.125, 0.5, 1, 1.5, and 2) (Figure 3). 

The energy fluctuation reduced with increasing Li/Na loading in both Li_2*x*_V_4_C_3_ and Na_2*x*_V_4_C_3_. However, the energy remained stable without any significant change over time, as illustrated in the straight line (Figure 3). That serves as an indication of the insignificant change in the structures of Li_2*x*_V_4_C_3_ and Na_2*x*_V_4_C_3_ without any deformations during Li/Na intercalation on the time scale of 1 fs to 5000 fs, which is in line with other reports on 2D materials [52,53,54]. We executed our simulations up to 5 ps (5000 fs) at 300 K. These steps are enough as the structure is retained at the end of 5 ps. It is noticed that the total energy converges right after as the time duration increases. Furthermore, our results show a low energy fluctuation.

### 3.3. Voltage and Li/Na Storage Capacity

**Figure 4:**
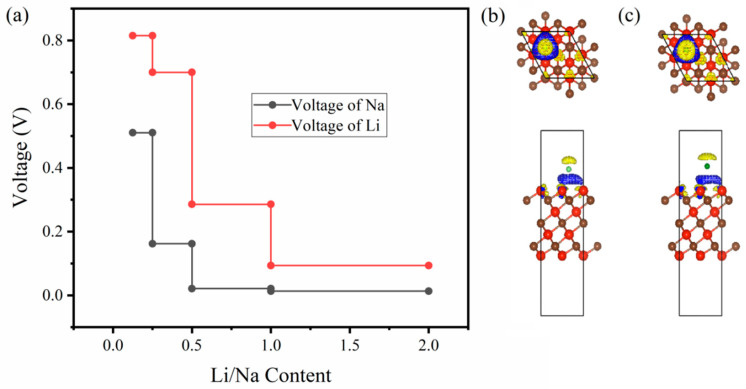
(**a**) Voltage plots of Li/Na. Charge density difference with front and side views of (**b**) Li adsorbed on site-1 and (**c**) Na loaded on site-1. The yellow color in (**b**,**c**) represents the electron deficit, blue is the accumulated electrons, red is V, and brown is C.

To further examine the electrochemical behavior of V_4_C_3_ as a Li/Na host for LIBs and SIBs, we calculated the open-circuit voltage (OCV). Here, we discuss the anodic behavior of V_4_C_3_ for both LIBs and SIBs. During the lithiation and delithiation processes, the anode reaction is indicated by V_4_C_3_ + *x*Li^+^ + *x*e^−^ ⇄ Li*_x_*V_4_C_3_. In this reaction, the charges (positive) start the motion between electrolyte and electrodes while the electrons pursue their motion through the external circuit of the cell. Ignoring the impact of temperature, pressure, and entropy, the voltage profile for Li/Na-loaded V_4_C_3_ is plotted in Figure 4a. Since the voltage profile depends on the binding energy, it decreases with the increase in Li/Na loading. However, our average voltages are estimated at around 0.38 V and 0.14 V for LIBs and SIBs. The computed voltages are underneath the described voltages of monolayers with Li/Na adsorption, where Li*_x_*SnC is 0.44 V, Li*_x_*Si_2_H_2_ is 0.42 V, Na*_x_*Si_2_H_2_ is 0.64 V, Na*_x_*SnS_2_ is 1.0 V, and Na*_x_*SnSe_2_ is 0.68 V [17,55,56]. Furthermore, our evaluated average voltages also satisfy the commercial anode materials (i.e., 0.11 V for graphite and 1.5–1.8 V for TiO_2_) [57,58]. Therefore, the suitable OCV designates the monolayer V_4_C_3_ as the superior Li/Na host material for LIBs and SIBs. Additionally, the amount of charge transfer is confirmed by evaluating the charge density difference as shown in Figure 4b,c for Li and Na, respectively. The isosurface marked with yellow exhibits the electron deficit, whilst the blue isosurface indicates the accumulated electrons. The results showed the possible charge transfer from Li/Na to the V_4_C_3_ surface and subsequently probable electrochemical reaction may occur between Li/Na and V_4_C_3_ [47,48,49]. 

The Li/Na storage capacity of 2D V_4_C_3_ is computed by employing the formula [59], *C* **=** *x*F/$M_{V_{4}C_{3}}$. In this equation, the terms *x*, F, and $M_{V_{4}C_{3}}$ define the Li/Na content loaded on V_4_C_3_, the Faraday constant possesses a noted value of 26,801 mAh mol^−1^, and the molar mass is per formula unit V_4_C_3_, correspondingly. According to the above formula, the Li/Na storage capacity is 223.5 mAhg^−1^ with a maximum loading of Li/Na content (*x* = 2). 

### 3.4. Li/Na Activation Energy Barriers

**Figure 5:**
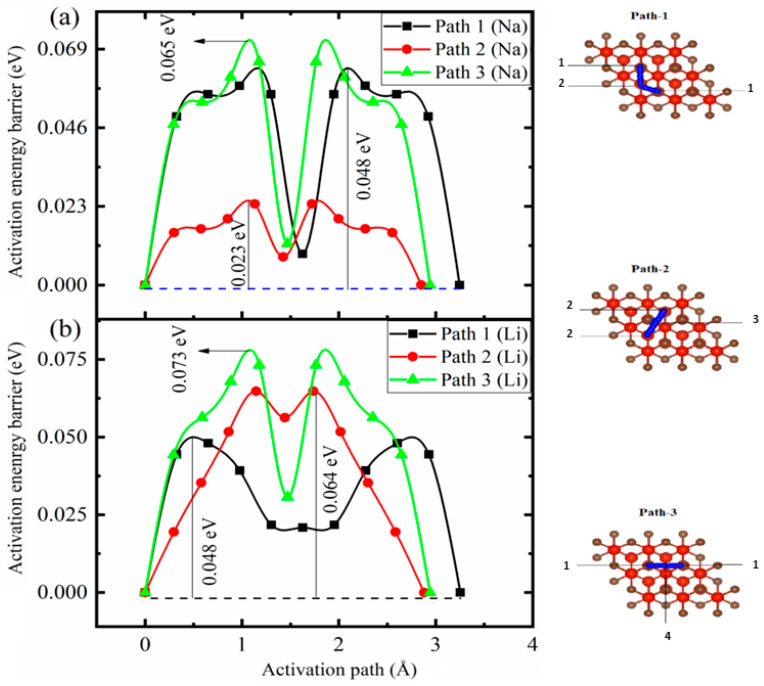
Activation pathways with their corresponding energy barriers of Na (**a**) and Li (**b**). The numbers (1-2-1, 2-3-2, and 1-4-1) represent the energy paths for the migration of Li/Na content (*x* = 0.0625).

In an electrochemical cell, the fast transportation of electrons and ions is desirable in a rechargeable battery to reduce the charging and discharging time. It is necessary to diffuse the metal ion at a rapid rate as it depends on the rate capability of the battery. To investigate the energy surface of V_4_C_3_ with Li/Na loading, we adopted a technique recognized as the climbing image nudged elastic band (CI-NEB) technique. This method is useful for finding the activation barriers and the corresponding paths. In the case of the monolayer V_4_C_3_ (2 × 2 × 1 supercell), we selected three minimum energy paths (MEPs), path-I (1-2-1), path-II (2-3-2), and path-III (1-4-1), for the migration of Li/Na content (*x* = 0.0625) as depicted in Figure 5. Five images are incorporated between the final and initial sites for each path. The simulated activation barriers for Li migration along the three pathways are 0.048 eV (path-I), 0.064 eV (path-II), and 0.073 eV (path-III). For Na migration, the computed diffusion energy barriers along the three paths are 0.048 (path-I), 0.023 eV (path-II), and 0.065 eV (path-III). The comparison of the results was made with the prior attempts, such as with Li*_x_*MoN_2_ (0.49 eV), Na*_x_*MoN_2_ (0.56 eV), Li*_x_*VN_2_ (0.237 eV), Na*_x_*CP_3_ (0.356 eV), and Li*_x_*B_3_S (0.32 eV). The MXene (V_4_C_3_) is dominant over other 2D materials due to its high Li/Na charging-discharging rates and low activation barriers. Moreover, we compared the diffusivity and voltages with some well-known anodes, as depicted in Table 2. The simulated results predict low diffusion energy barriers for Li/Na on V_4_C_3_ compared to graphitic materials (0.277~0.47 eV) [60,61], illustrating an enhanced rate capability of the host (V_4_C_3_) for LIBs and SIBs.

### 3.5. Electronic Properties

**Figure 6:**
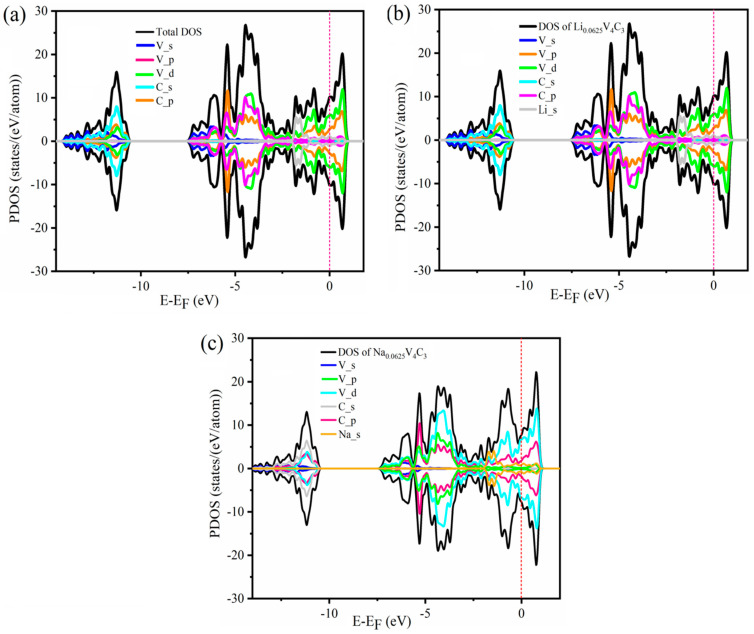
Density of states of (**a**) bare V_4_C_3_, (**b**) Li_0.0625_V_4_C_3_, and (**c**) Na_0.0625_V_4_C_3_.

Besides electronic conductivity, another essential attribute of anode materials is their superior performance. This can be assessed thoroughly to study the electronic structure, such as the density of states (DOS). Therefore, we performed the GGA-PBE calculations to establish the density of states (DOS) and partial density of states (PDOS) of pristine V_4_C_3_ *MX*ene and Li/Na (*x* = 0.0625)-loaded V_4_C_3_ (Figure 6). Employing the GGA-PBE technique, the DOS of the monolayer V_4_C_3_ was expected to be of a possible metallic nature (Figure 6a). The metallic character of the bare V_4_C_3_ was further examined by PDOS. The main contributions occur due to the state of V_d and C_p in the conduction band. However, the other states show small contributions to electronic conductivity. The states, such as V_p and C_s, mainly contribute to the valence band. These results justify the initial efforts made on electronic structures of the V_4_C_3_ [46]. 

The PDOS is depicted in Figure 5b,c after loading the Li/Na content on the supercell of V_4_C_3_ at an insignificant amount (*x* = 0.0625). Furthermore, the electronic structures of Li/Na-loaded V_4_C_3_ are inspected at *x* = 0.0625. At low Li/Na loading, the metallicity of the material is still maintained (i.e., Li_s or Na_s). Thus, the charge carrier transfer to the conduction band is predicted to improve electronic conductivity. The enhanced electronic conductivity suggests the better performance of V_4_C_3_ as an outstanding host material for both LIBs and SIBs. 

## 4. Conclusions

In summary, a first-principle DFT simulation was utilized to predict the performance of V_4_C_3_ MXene as an anode for LIBs and SIBs. To this end, the electronic properties, durability, voltage, storage capacity, and activation barriers of Li/Na-loaded V_4_C_3_ were assessed. The results displayed super performances of the Li_2*x*_V_4_C_3_ and Na_2*x*_V_4_C_3_ as anodes for LIBs and SIBs, with an average potential of 0.38 V (for Li) and 0.14 V (for Na), as well as a reasonable Li/Na storage capacity of 223 mAhg^−1^ and good cycle performance. In addition, V_4_C_3_ reveals very low diffusion energy barriers of 0.048 eV (for LIBs) and 0.023 eV (for SIBs), indicating the possible fast lithiation/delithiation and sodiation/desodiation processes. As the Li/Na content increased, the voltage decreased from 0.8 to 0.1 V for Li V_4_C_3_ and from 0.5 to 0.05 V for NaV_4_C_3_. During Li and Na intercalation, the energy fluctuation vs. time duration revealed a straight line, implying structural stability without any apparent deformations. The process also stems from the prompt recovery of V_4_C_3_, structure stability during Li/Na, and ion intercalation/extraction. The presented findings may create the opportunity for further usage of V_4_C_3_ as an anode material for LIBs and SIBs.

## Figures and Tables

**Table 1:** Structural parameters of pristine V_4_C_3_ M*X*ene and Li/Na content-loaded V_4_C_3_ (2 × 2 × 1 supercell) at *x* = 0.0625, including binding energy and charge transfer.

Parameters	Simulated Values			
*E*ad (eV) for Li	1-site	2-site	3-site	4-site
	0.90	0.884	0.828	0.897
*E*ad (eV) for Na	1.21	1.16	1.15	1.20
Charge *q* (|e|) for Li	0.84	0.83	0.83	0.883
Charge *q* (|e|) for Na	0.67	0.664	0.66	0.665
Height (h_S-S_)	6.96 Å			
Lattice constants (*a*, *b*)	2.90 Å			

**Table 2:** Comparison of voltages and energy barriers with Li*_x_*V_4_C_3_ and Na*_x_*V_4_C_3_.

Material	Voltage	Diffusion Barrier Energy		Reference
Na*_x_*MoS_2_	0.56 V	0.08 eV	Method	[62]
			NEB	
Na*_x_*W_2_C	0.43 V	0.019 eV	NEB	[59]
Na*_x_*SiS	0.10 V	0.18 eV	CI-NEB	[63]
Li*_x_*WSe_2_	0.67 V	0.24 eV	NEB	[64]
Li*_x_*SiH	0.42 V	0.18	CI-NEB	[56]
2D K*_x_*PC	0.69 V	0.26 eV	NEB	[65]
2D K*_x_*SnC	0.41 V	0.17 eV	NEB	[66]
3D Li*_x_*PBC_2_	0.48 V	0.29 eV	CI-NEB	[67]
3D Li*_x_*Si_2_BN	0.27 V	0.44 eV	NEB	[68]
Li*_x_*V_4_C_3_	0.38 V	0.048 eV	CI-NEB	This work
Na*_x_*V_4_C_3_	0.14 V	0.023 eV	CI-NEB	This work

## Data Availability

The data presented in this study are available on request from the corresponding author.

## Supplementary Materials

The following supporting information can be downloaded at: https://www.mdpi.com/article/10.3390/nano12162825/s1, Figure S1: Side views of various models of Li/Na loaded on V_4_C_3_ monolayer, Figure S2: Variation of lattice parameters with increasing Li/Na content, and Voltage profile.
