# Supplementary material for: Vanadium Carbide (V4C3) MXene as an Efficient Anode for Li-Ion and Na-Ion Batteries
Source: Nanomaterials (Basel). 2022 Aug 17;12(16):2825. doi: 10.3390/nano12162825 (PMC9416528; doi:10.3390/nano12162825)
Supplement: Supplementary file 1 [file nanomaterials-12-02825-s001.zip › nanomaterials-1769324-supplementary.pdf]

nanomaterials

MDPI

Supplementary Materials

Vanadium Carbide (V_4C_3) MXene as an Efficient Anode for Li-Ion and Na-Ion Batteries

Qiong Peng [1], Javed Rehman [2,*], Kamel Eid [3,*], Ayman S. Alofi [4], Amel Laref [5], Munirah D. Albaqami [6], Reham Ghazi Alotabi [6] and Mohamed F. Shibl [7]

1 Istitution of Condensed Physics & College of Physics and Electronics Engineering, Hengyang Normal University, Hengyang, 421002, China
2 Department of Physics, Balochistan University of Information Technology, Engineering and Management Sciences (BUITEMS), Quetta 87300, Baluchistan, Pakistan
3 Gas Processing Center (G.P.C.), College of Engineering, Qatar University, Doha 2713, Qatar
4 Physics Department, College of Science, Taibah University, Medina 42353, Saudi Arabia
5 Department of Physics and Astronomy, College of Science, King Saud University, Riyadh 11451, Saudi Arabia
6 Chemistry Department, College of Science, King Saud University, Riyadh 11451, Saudi Arabia
7 Center for Sustainable Development, College of Arts and Sciences, Qatar University, Doha 2713, Qatar
* Correspondence: javed.rehman@buitms.edu.pk (J.R.); kamel.eid@qu.edu.qa (K.E.)

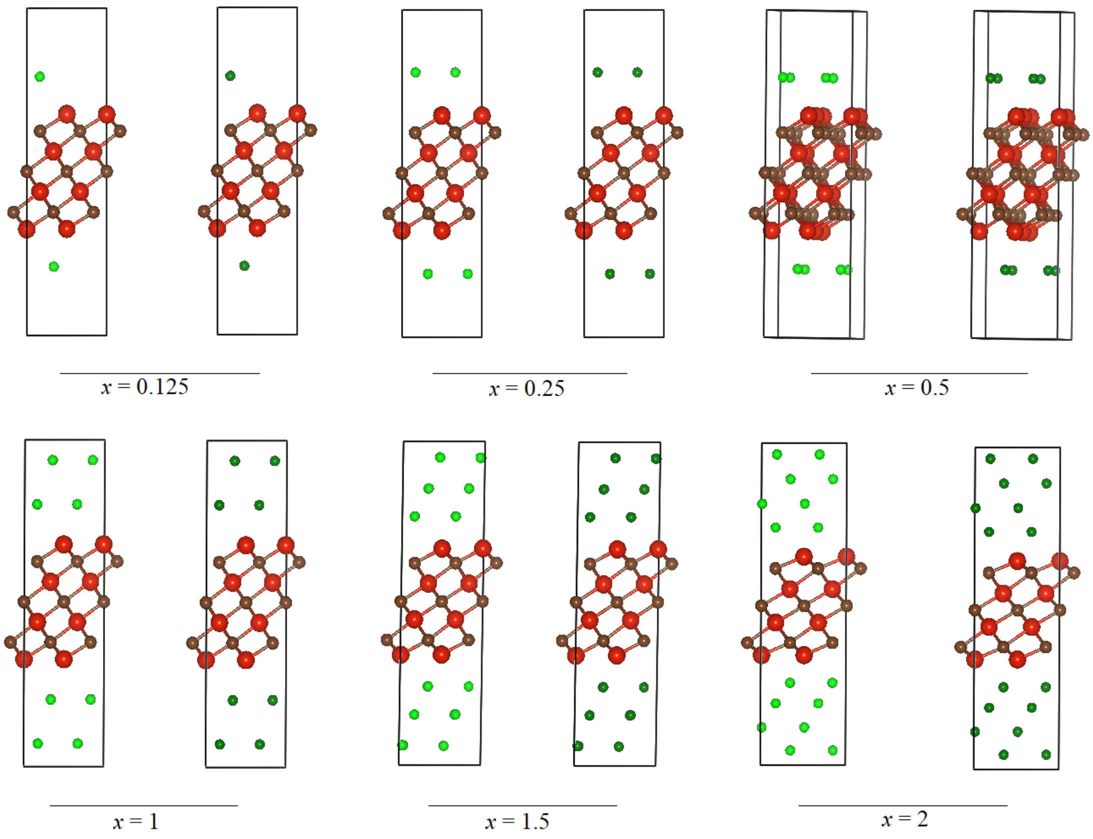

Figure S1. Side views of various models of Li/Na loaded on V_4C_3 monolayer.

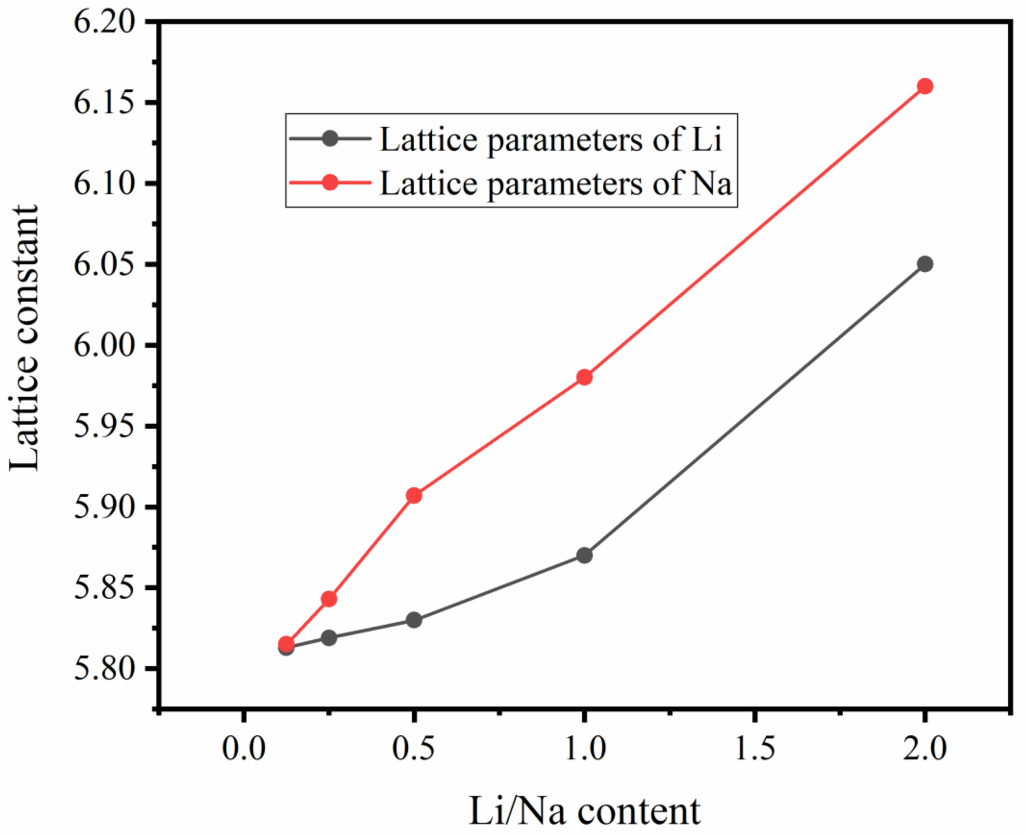

Figure S2. Variation of lattice parameters with increasing Li/Na content.

Voltage profile: The equilibrium voltage of Li intercalation is calculated by the difference in chemical potential of Li (μ_{Li}) between anode and cathode, i.e.

$$V = [-(\mu_{Li})^{cathode} - (\mu_{Li})^{anode}]/zF,$$

where F is the Faraday constant, and z is the charge transferred. With Li content, the μ_{Li} is the change of the free energy of the electrode material. By integrating the above equation over a finite amount of reaction, one can obtain the average voltage as a function of the free energy change of the combined anode/cathode reaction (Nernst equation). Thus, the voltage is expressed as $V = -\Delta G_r/zF$.

The entropic contributions to ΔG_r are negligible at a low temperature, and the reaction free energy is approximately equal to the internal energy ($\Delta G_r \approx \Delta E_r$). Within this approximation, we can analyze the voltage profile by the results of the first principle calculation. For example, the equilibrium voltage of lithium intercalation cathode with composition $LiMO_2$ and a lithium metal anode with the cell reaction is given by,

$$Li_{x1}\,MO_2 \longrightarrow Li_{x2}\,MO_2 + (x_1 - x_2)Li$$

and

$$V(x_1, x_2) = [E_{Li_{x1}MO_2} - E_{Li_{x2}MO_2} + (x_2 - x_1)\,E_{Li}]/(x_2 - x_1)e, \text{ with } x_2 > x_1,$$

where $E_{Li_{x1}MO_2}$ and $E_{Li_{x2}MO_2}$ are the energies of the $Li_{x1}\,MO_2$ and $Li_{x2}\,MO_2$, and E_{Li} is the energy of bulk Li. These can be predicted from the first principles.
